# New Test for Arsenic

**Published:** 1865-11

**Authors:** 


New Test for Arsenic.—The wounderful delicacy of the previous tests for arsenic which have been thought to be almost perfect, are surpassed by the electrical test. By means of a simple apparatus all the arsenic in a substance may be rapidly extracted. Place a solution of arsenic matter in a platinum vessel, plunge a zinc wire into the liquid, and the arsenic will appear on the plantinum; by prolonging the action the whole of the arsenic may be extracted from the compound. This process is superior in sensibility, and as it requires far less manipulation of the suspected substance, is much more trustworthy for toxicological examinations than the methods now in use.

